# Predicting extubation failure

**DOI:** 10.1186/2197-425X-3-S1-A996

**Published:** 2015-10-01

**Authors:** S Krivinskas, R Sarkar, O Turner, K Goonetilleke, P Anderson

**Affiliations:** Royal Sussex County Hospital, Intensive Care, Brighton, United Kingdom; Royal Sussex County Hospital, Brighton, United Kingdom

## Introduction

Predicting extubation failure in Intensive Care is vital to reduce mortality and lower costs due to increased length of Intensive Care Unit (ICU) and hospital stay. We look at predictors of extubation failure in patients passing a spontaneous breathing trial.

## Objectives

To identify specific factors leading to increased risk of failed extubation.

## Methods

A retrospective study was conducted of patients who had been re-intubated within 72 hours of extubation at the Royal Sussex County ICU between Dec'02 and Nov'14. Only patients who were intubated intially within the unit were included as they could be identified by the set search criteria in the electronic system.

## Results

Out of 680 intubations, 73(11%) patients [46 (63%) male] had failed extubations. Average age was 60(21-88)years. Average time to re-intubation was 22.1(1-72) hours, 45(62%) being re-intubated at≤24 hours. Average time intubated was 2.2 days with 3 patients intubated for >7 days.

Fifteen(21%) patients had had no sedation within 4 hours of extubation. All others had one or more of fentanyl, Remifentanil, propofol, midazolam, and morphine. Eighteen (25%) patients had a Glasgow Coma score(GCS)< 10(eyes 4, voice 1, motor 5), of which 16(89%) were on at least one sedative. Five patients did not have GCS recorded. Forty-four (60%) patients had mucoid secretions, the rest having thick, purulent or yellow secretions.

Mean respiratory rate(RR) was 19/minute (range 9-39) with mean rapid shallow breathing index (RSBI) f/Vt 37.5 breaths/min/l (10.8 - 203.1). Eight (11%) patients had FiO2 >40% pre-extubation. Mean pre-extubation PEEP was 5.8cmH2O(5-10) with mean pressure support(PS) of 14cmH2O (range 5-27), with 57(78%) having PS >10.

Fifty eight(80%) patients were hypercapnoeic(PaCO2 >45mmHg) pre-extubation, 2 patients had no arterial blood gas documented shortly prior to extubation, 1 with venous sample. Of these 58 patients, 5 (9%) were acidotic and 8 (14%) received NIV (6 CPAP, 2 BIPAP) immediately post extubation. One patient self-extubated. Post extubation ABG showed 15/58 (26%) had respiratory acidosis (PH < 7.35, PaCO2 ≥ 60mmHg), 3 of which received NIV immediately post extubation.

## Conclusions

Incidence of failed extubation was low at 11%. Failed extubation happened across all age groups and almost half(45%) the patients needed re-intubation within the first 24 hours. Low GCS may increase risk but may also be due to lack of sedation wean pre-extubation.

RSBI was not a good indicator at predicting risk as only one patient scored >105.

High PS (mean 14cmH2O) pre-extubation very likely contributed towards extubation failure. Hypercapnoea pre-extubation is a risk factor for failed extubation and use of NIV post extubation was low at 14% in these patients. Use of NIV in this group possibly could have averted re-intubation [1].
